# Deterministic Lateral Displacement Microfluidic Chip for Minicell Purification

**DOI:** 10.3390/mi13030365

**Published:** 2022-02-25

**Authors:** Ahmad Sherbaz, Büşra M. K. Konak, Pegah Pezeshkpour, Barbara Di Ventura, Bastian E. Rapp

**Affiliations:** 1 Laboratory of Process Technology, NeptunLab, Department of Microsystems Engineering (IMTEK), University of Freiburg, 79110 Freiburg im Breisgau, Germany; ahmad.sherbaz@neptunlab.org (A.S.); bastian.rapp@neptunlab.org (B.E.R.); 2 Signalling Research Centres BIOSS and CIBSS, Institute of Biology II, Faculty of Biology, University of Freiburg, 79110 Freiburg im Breisgau, Germany; busra.merve.kirpat.konak@biologie.uni-freiburg.de (B.M.K.K.); barbara.diventura@biologie.uni-freiburg.de (B.D.V.); 3 Freiburg Materials Research Center (FMF), University of Freiburg, 79104 Freiburg im Breisgau, Germany; 4 FIT Freiburg Center of Interactive Materials and Bioinspired Technologies, University of Freiburg, 79110 Freiburg im Breisgau, Germany

**Keywords:** deterministic lateral displacement, microfluidics, bacterial cell separation, minicells, *Escherichia coli*

## Abstract

Deterministic lateral displacement (DLD) is a well-known microfluidic technique for particle separation with high potential for integration into bioreactors for therapeutic applications. Separation is based on the interaction of suspended particles in a liquid flowing through an array of microposts under low Reynolds conditions. This technique has been used previously to separate living cells of different sizes but similar shapes. Here, we present a DLD microchip to separate rod-shaped bacterial cells up to 10 µm from submicron spherical minicells. We designed two microchips with 50 and 25 µm cylindrical posts and spacing of 15 and 2.5 µm, respectively. Soft lithography was used to fabricate polydimethylsiloxane (PDMS) chips, which were assessed at different flow rates for their separation potential. The results showed negligible shear effect on the separation efficiency for both designs. However, the higher flow rates resulted in faster separation. We optimized the geometrical parameters including the shape, size, angle and critical radii of the posts and the width and depth of the channel as well as the number of arrays to achieve separation efficiency as high as 75.5% on a single-stage separation. These results pave the way for high-throughput separation and purification modules with the potential of direct integration into bioreactors.

## 1. Introduction

Deterministic lateral displacement (DLD) post arrays are an efficient tool to separate and enrich micrometer-scale particles, such as parasites, bacteria, blood cells and circulating tumor cells in blood [1,2,3,4]. Discovered 50 years ago, minicells, spherical particles arising from aberrant asymmetric bacterial cell division events, have recently been exploited as model systems to visualize molecular machines in situ, due to their smaller size and other unique properties [5]. They provide a springboard for in-depth structural studies of bacterial macromolecular complexes and therefore offer a unique approach for gaining novel structural insights into many important processes in microbiology [6]. Like their parental cells, they contain membranes, RNAs and proteins but no chromosome; therefore, they cannot divide or grow. There is growing interest in developing minicells for drug delivery [7,8], cancer therapy [4] and antigen delivery for vaccine development [9]. Several techniques are involved in minicell purification including biochemical methods, by using reagents to eliminate the parental cells, or sized-based methods including centrifugation, sonication and size-exclusion chromatography [4,10] or hydrodynamic chromatography [11]. In chromatography, particles pass through a pore-structured tunnel and separate based on their size. Hydrodynamic techniques make use of particle movement along the hydrodynamic parabolic velocity profile in capillary tubes. Large particles cannot intercept the low-velocity fluid near the capillary wall; thus, on average, they move faster and become separated from small particles [12]. Based on the hydrodynamic rules, initiated from the Austin group and in collaboration with the Sturm group at Princeton, DLD was introduced as a microfluidics-based particle separation technique, where particles are separated based on their size by flowing through an array of posts on a microchip [4,5]. In a DLD device, the total fluid flux through each gap between microfabricated post arrays is divided into flow streams where each flow stream carries equal fluid flux. The streamlines are separated by stall lines, beginning and ending on a post. The streamlines shift their positions cyclically so that after a few rows each streamline returns to its initial position within the gap. If a particle’s radius is larger than the width of the first streamline, the particle will be forced to remain in the second or higher-numbered streamline in every row. It will be “bumped” deterministically by each subsequent row, thus traveling in the so-called “bump mode”. If a particle’s radius is smaller than the width of the first streamline, it will follow the cyclic procession of the streamlines and travel in the so-called “zigzag” mode. The critical particle radius is the dividing line between the two modes of travel and, in this model, is equal to the width of the first streamline. The parameter of interest is the critical particle diameter, which is twice the width of the first streamline [13]. This work was later extended introducing cylindrical and triangular post arrays [14]. The geometry of the posts was thoroughly studied to optimize the effect of pillar shape on the separation of cells with two morphologies [15].

Most work on DLD has, so far, explored the separation of cells with less than one order of magnitude difference in size. Holm and colleagues reported a DLD microchip for separating red blood cells from living parasites exploring differences in diameter with a factor of 4 [3]. Liu and colleagues presented high-throughput isolation of cancer cells using DLD [16]. For the first time, Wunsch and colleagues implemented DLD microchips for the separation of nanometer-scale exosomes functioning as biocolloids [17]. One-dimensional Brownian motion on microchips with arrays of rachets has also been implemented to separate short DNA fragments based on diffusion separation techniques [18]. Two-dimensional numerical simulations were carried out on the geometry of post arrays to study the effect of the pillar shape, row shift and pillar diameter on the performance of red blood cell separation [1]. The design was optimized by providing 6 µm circular posts with low shift angle. In another work, the effects of cell size and morphology as well as chip geometry were studied and the separation of deformable red blood cells down to 2 µm diameter was achieved [19]. With scaled-up microchips in stackable fashion, 0.73% to 88% recovery of chimeric antigen receptor (CAR) T cells was reported [20]. In recent years, DLD has been implemented for a variety of applications such as separation of fetal cells in noninvasive prenatal testing [21] as well as an immunoaffinity method based on antibody-mediated antigen enrichment [22]. To increase the separation efficiency, external electric fields, e.g., in dielectrophoresis, can be combined with DLD to better tune the trajectory of the particles [22,23]. Shirenji and colleagues compared different microfluidic approaches relying on inertial lift force, viscoelastic flow, acoustic waves and dielectrophoresis combined with DLD as a low-cost platform [24]. These approaches were mostly tailored for blood cell separation with no requirement for external forces or additives. 

In this work, we present a DLD device capable of separating *Escherichia coli* bacterial cells based on size and shape without the use of external forces. Unlike DLD separation microchips reported so far, the cells in the present work are not only of different size, but also have different geometries, namely spherical versus rod-shaped. Rod-shaped parental cells are in the range of up to a few tens of microns and are separated from submicron spherical minicells with high efficiency on the presented single-stage DLD chip. 

## 2. Materials and Methods

### 2.1. Materials for DLD Microchip and Cell Solution

The materials used in the DLD microfluidic chip fabrication include silicon wafer (Clean Room Service Centre IMTEK, Germany), photoresist SU-8 3025 (Micro-resist Technology, Berlin, Germany), ethyl L-lactate purum (Sigma-Aldrich, Taufkirchen, Germany), polydimethylsiloxane (PDMS) (RT-601, WACKER, Munich, Germany), 180 µm-thick glass coverslip (Ibidi GmbH, Graefelfing, Germany) and isopropanol (Roth, Karlsruhe, Germany). For cell culture, we used LB medium (Roth, Karlsruhe, Germany), disposable 20 mL syringe Omnifix with Luer-Lock fitting (Roth, Karlsruhe, Germany), Epredia coverslip 18 mm × 18 mm (0.08–0.12 mm, Thermo Fisher scientific, Schwerte, Germany), Menzel Gläser microscope slides 76 mm × 26 mm (Thermo Fisher scientific, Schwerte, Germany), agarose (Sigma-Aldrich, Taufkirchen, Germany), kanamycin (Sigma-Aldrich, Taufkirchen, Germany), ampicillin (Sigma-Aldrich, Taufkirchen, Germany), chloramphenicol (Sigma-Aldrich, Taufkirchen, Germany) and L- (Sigma-Aldrich, Taufkirchen, Germany). 

### 2.2. Chip Fabrication

DLD microchips were fabricated by soft lithography [25,26]. Briefly, 25 mm disc-shaped silicon (Si) wafers were cleaned with acetone for 10 s. The wafers were spin-coated with 1 mL photoresist SU-8 3025 at 500 rpm with acceleration of 100 rpm/s for 10 s and further at 3000 rpm with acceleration of 300 rpm/s for 30 s. After spin-coating, the wafer was soft-baked at 95° for 15 min before exposing the photoresist (350 nm, 50 mJ/cm^2^, 30 s, mask aligner HiRes-Koenen, Ottobrunn-Riemerling, Germany) and postexposure baking (95°, 5 min). The wafer was then immersion-developed in ethyl lactate for 5 min before rinsing with isopropanol and distilled water followed by drying using pressurized air. The thus created replication master structure was then replicated using PDMS to the final microfluidic chip structure. For this, PDMS precursors A and B were mixed in a 10:1 ratio (by weight) according to the manufacturer’s specification. The mixture was degassed using a desiccator before pouring onto the replication template. The solution was cured in an oven at 65° for 24 h. Inlets and outlets were punched by a mechanical drill of 1.2 mm diameter. To close the chip, PDMS was bonded to a coverslip glass with 180 µm thickness using an air plasma system (Zepto, DIENER, Ebhausen, Germany) at 1 mbar pressure for 1 min. The chip was fluidically connected to an external syringe pump (Legato 100, KDS, Germany) using Teflon tubing (inner diameter 1 mm).

### 2.3. Cell Solution Preparation

The cell solution was prepared as described elsewhere [27,28]. In short, chemically competent *E. coli* MG1655 ∆*minB* cells were transformed with plasmid pUC19-pLacO-sfGFP (without LacI). The cells constitutively express the superfolder green fluorescent protein (sfGFP), which allows tracking them in a fluorescence microscope. A glycerol stock with the cells bearing the plasmid was generated. An overnight culture was started by inoculating cells from the glycerol stock into Luria-Bertani (LB) medium (tryptone 10 g, yeast extract 5 g, NaCl 10 g for 1 L) and placing it in an incubator shaker at 37 °C. The next day, the culture was either used directly in the chip or used to purify minicells. To purify minicells, the overnight culture was diluted 1:1000 (by volume) into 1 L of LB and incubated for 24 h at 37 °C at 200 rpm. Afterward, the culture was centrifuged two times at 4000× *g* and 4 °C for 10 min to eliminate the rod-shaped parental cells. Minicells were pelleted by centrifugation (20,000× *g*, 4 °C for 20 min), and then the pellet was resuspended in 100 mL of LB medium. To kill actively dividing parental cells, the minicell solution was treated with 100 µg/mL ceftriaxone at 37 °C and 200 rpm for 2 h. After antibiotic treatment, the culture was again centrifuged (4000× *g*, 4 °C for 10 min) to discard remaining parental cells and cell debris. The supernatant was recentrifuged (20,000× *g*, 4 °C for 20 min) to concentrate minicells and the pellet was resuspended in 10 mL of LB. The *E. coli* MG1655 wild-type strain containing plasmid pBAD33-mCherry was additionally used as control for cells of normal length. These cells express the red fluorescent protein mCherry under L-arabinose induction and can thus be tracked using a fluorescence microscope. The overnight culture of this strain was diluted 1:50 (by volume) in 20 mL of LB and grown at 37 °C with shaking at 200 rpm until OD_600_ reached 0.4, and mCherry protein expression was induced with 0.1% L-arabinose.

Throughout this work, two types of solutions were used: (1) a mixed solution, which was closest to the actual mixture within the bioreactor and consisted of parental cells (1 to 10 µm, rod-shaped cells) and minicells (spherical cells with diameter in the range of 400 to 500 nm), and (2) wild-type solution, which refers to normal-sized cells, not mixed with any minicells. 

### 2.4. Fluorescence Microscopy

Fluorescence microscopy was performed on a Zeiss Axio Observer Z1/7 (Carl Zeiss, Germany) inverted wide-field microscope, equipped with a Colibri 7 LED light source, an Alpha Plan-Apochromat × 100/1.46 oil DIC (UV) M27 objective, filter sets 38 HE (ex. 450–490, dichroic beam splitter 495, em. 500–550; sfGFP), 43 HE (ex. 538–562, beam splitter 570, em. 570–640; mCherry) and an Axiocam 506 Mono camera. Samples were embedded onto 1% agarose pads on metal microscopy slides prior to imaging. Cell numbers were counted manually from the images.

### 2.5. DLD Chip Design

**Figure 1:**
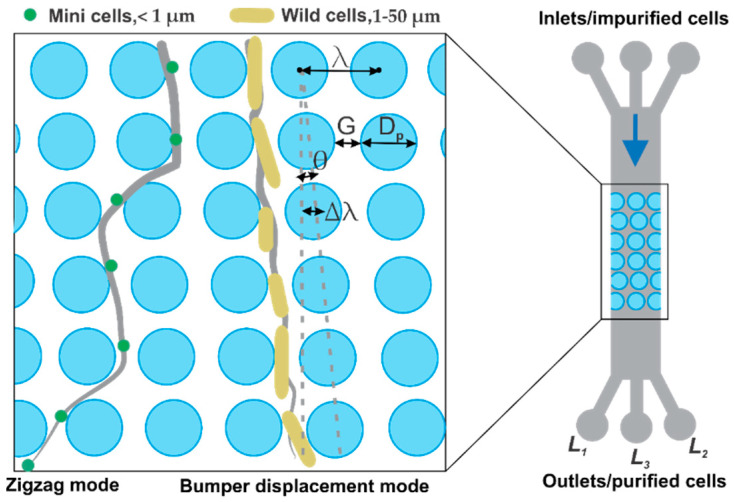
Schematic of the deterministic lateral displacement (DLD) microchip for separation of *E. coli* minicells (spherical) and parental (rod-shaped) cells.

The DLD microfluidic chip in Figure 1 shows the concept of cell separation for two types of cells based on different sizes and shapes. The smaller cells follow the zigzag mode while the larger ones follow the bump mode displacement towards the channel walls. 

The design parameters of the DLD bumper array include the following:

(1) $$\epsilon =\frac{\Delta \lambda }{\lambda }\;$$

(2) $$\lambda =G+D_{P}$$

(3) ϵ=tanθ

(4) $$D_{c}=1.4G\epsilon^{0.48}$$

ε is the tangent of the angle between each period shifted with respect to the previous one [14]. λ represents the constant center-to-center distance between two posts which is also the sum of the gap distance G and post diameter $D_{p}$. The critical diameter $D_{c}$ is calculated from Equation (4). The details of chip designs are presented in Table 1. Chip A was designed as a control experiment with D_c_ set to zero in order to observe the morphology and size effects of the cells and comparatively calculate the efficiency of the optimized Chip B. Given the range of sizes for different shapes of the cells in this work, Chip B was revised with shifted rows which allow only minicells in zigzag mode whereas all other cells follow a bump mode. 

Based on these parameters, two designs with $D_{p}$ = 50 and 25 µm and G = 15 and 2.5 µm, respectively, were implemented. Chip A included 50 µm posts arrayed with 0° shift whereas Chip B had 25 µm posts which were shifted by 1°. The main channel including the array of posts was 50 mm × 1.2 mm with 3 inlets and outlets (each 3.2 mm in diameter) which were connected via 5 mm × 0.4 mm channels to the main channel. The middle inlet served as the entry point of the cell suspension prior to the separation process with two flanking inlets acting as control inlets for the suspension, whereas the separation efficiency could be observed via the three outlets L_1_, L_2_ and L_3_. However, during the experiments, the flanking inlets were blocked as no further sheath flow control was required. The numbers of minicells and parental cells were counted in 1 mL of the impurified solution and were compared with the counts of the sample extracted from outlets L_1_, L_2_ and L_3_ after purification, respectively. Following this observation, the efficiency of each design was calculated by separation efficiency introduced in Equation (5).

**Figure 2:**
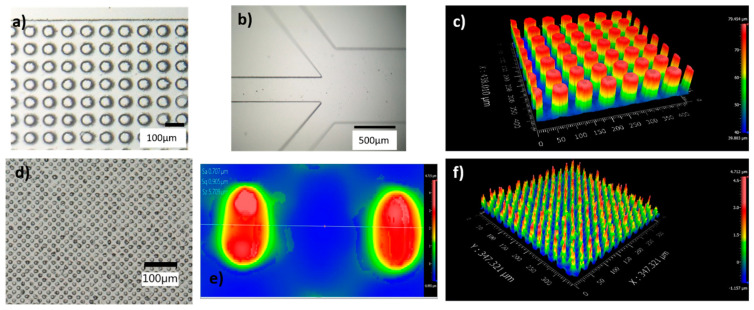
Fabricated DLD PDMS chips. (**a**) Array of 50 µm posts with 0° row shift (Chip A), (**b**) side channels at inlet/outlet, (**c**) white light interferometry of 50 µm posts (Chip A), (**d**) array of 25 µm posts (Chip B). (**e**,**f**) White light interferometry of 25 µm posts (Chip B).

The fabricated posts and side channels are shown in Figure 2. The posts are individually formed, and no defective area is observed. The posts are not tilted or stuck together. The side walls also show smooth surfaces and the corners of the side channels towards the outlets (Figure 2b) are nicely formed. In addition, the flow was assessed via fluorescence microscopy that showed uniformity across the entire channel from the inlet to the outlets, as shown in Figures S1 and S2. 

## 3. Results and Discussion

To separate minicells from parental cells in the bacterial cell solution, two sets of experiments were run with Chip A along with a corresponding control experiment. First, to assess how rod-shaped cells up to 10 µm long would be distributed in the chip, we used a culture with only wild-type cells and monitored their accumulation at the outlets. Second, a mixed solution consisting of both parental and minicells was used to assess the efficiency of the designed chip and the flow content. Moreover, the first experiment was carried out with Chip A to determine the morphology effects of the cells, while the second experiment allowed us to determine the size effects. 

**Figure 3:**
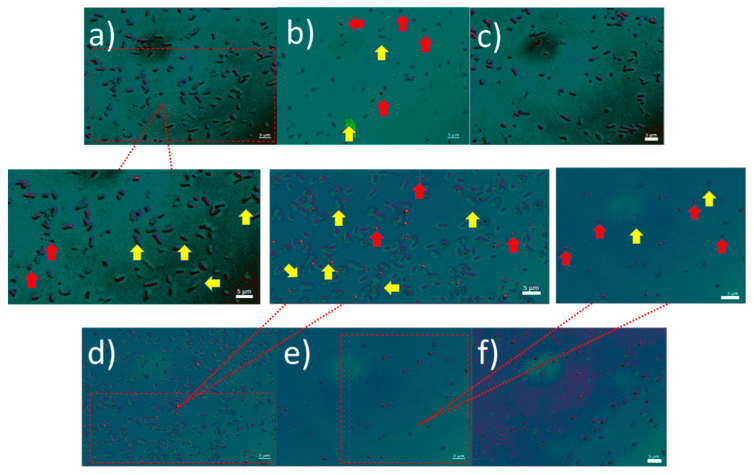
Fluorescence microscopy images of wild-type cells at 50 µL/min flow rate for Chip A at the L_1_ (**a**), L_3_ (**b**) and L_2_ (**c**) outlets and Chip B at the L_1_ (**d**), L_3_ (**e**) and L_2_ (**f**) outlets. The yellow arrows show the wild-type cells larger than 1 µm while the red arrows point to the wild-type cells that are equal to or smaller than 1 µm.

The tests were carried out using three distinct flow rates (10, 25 and 50 µL/min), all in the laminar flow regime with low Reynolds number (*Re* < 1), with the aim of assessing the effect of shear on the separation efficiency at higher flow rates (Videos S1 and S2). Higher flow rates allow for faster real-time purification, which would be advantageous in applications where throughput is critical. To clear the channels from residues, water was pumped for 2 to 4 min until a clear flow was observed. After pumping 5 mL of each solution, we measured the efficiency of separation using Equation (5) by sampling 1 µL of the initial solution before running through the DLD chip and after separation:

(5) $$\eta =1-{\left[\frac{{\left(\frac{\mathrm{parental}\;\mathrm{cells}}{\mathrm{mini}\;\mathrm{cells}\;}\right)}_{\mathrm{purified}\;\mathrm{solution}}}{{\left(\frac{\mathrm{parental}\;\mathrm{cells}}{\mathrm{mini}\;\mathrm{cells}\;}\right)}_{\mathrm{solution}\;\mathrm{before}\;\mathrm{separation}}}\right]}_{\;}$$

where η is the “separation efficiency” as the percentage of cell separation and is calculated by taking the ratio of parental cells over minicells in the purified solution divided by the same ratio in the solution prior to the separation, i.e., from the initial input cell suspension. We counted the number of parental cells and minicells per 1 mL of the initial input cell suspension before the purification and at the outlets, comparing outlet L_3_ to the other two outlets. The images taken from each outlet of Chips A and B can be seen in Figure 3. As per the flow profile, lesser elongated (parental) cells accumulate in the middle outlet (L_3_), where the minicells are mostly dominant. After creating a mixed solution where both types of cells were present, the input cell suspension was pumped to both microchips at three different flow rates (10, 25 and 50 µL/min). The results show that cells with a diameter above Dc flow in bumping mode, while the cells with a diameter smaller than Dc move following a zigzag path. To determine the efficiency of each chip, we first investigated the role of the morphology of the cells (rod-shaped), and then we looked into the role of cell size in separation.

### 3.1. Wild-Type Cell Flow (Morphology Effect)

**Figure 4:**
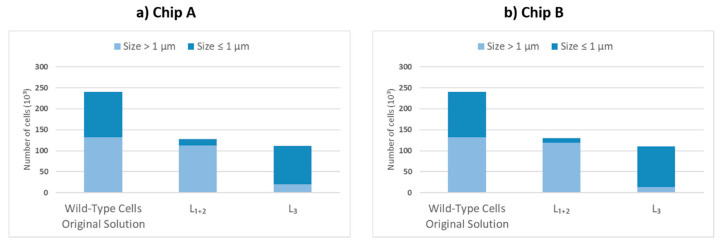
Number of wild-type cells in the solution prior to separation and the ratio of cells categorized by their size at the indicated outlets for (**a**) Chip A and (**b**) Chip B.

Unlike most of the previously described DLD microchips, we considered two parameters in the separation of the cells: shape and size. Moreover, the rod-shaped parental cells varied in size in the mixed solution. To assess the behavior of the wild-type cells, which effectively is a control experiment to observe the effects of size, we used a culture made of only wild-type cells as a sample solution and assessed the accumulation of the cells at the outlets. The solution prior to separation was analyzed, and the total number of wild-type cells present in 1 mL of the solution was estimated to be 240,000. We found that wild-type cells with a size larger than 1 µm were separated from those with a size equal to or less than 1 µm. The ratio between these two size ranges was found to be 45% of cells smaller than or equal to 1 µm in the solution prior to the separation. The separation was carried out with both chips. Chip A, having a critical diameter set to zero, yielded a post flow solution in L_3_ consisting of 85% of cells smaller than or equal to 1 µm in L_3_, as shown in Figure 4. When the same solution was tested with Chip B, the postseparation solution consisted of 90% of cells smaller than or equal to 1 µm in L_3_. However, since Chip B has a critical diameter of 500 nm, the total number of cells accumulated at the outlet L_3_ per 1 mL after separation drops by 5%, as shown in Figure 4. Chip B allows for the same separation as chip A, which imposes a certain effect on the flow of rod-shaped wild-type cells and pushes the larger cells to flow predominantly into the flanking outlets (L_1_ and L_2_), while the smaller cells maintain a straight flow into the middle outlet (L_3_). In conclusion, this experiment confirmed that size influences to some degree the flow profile of the wild-type, rod-shaped cells. Having a lattice shift with Chip B or a symmetric lattice does not modify the profile of the wild-type cells, whose size determines the flow profile. Moreover, the effects of size change when combined with morphology effect when it comes to having two differently shaped cell types, as is presented in the mixed solution experiment below.

### 3.2. Mixed Solution Flow (Size Effect)

**Figure 5:**
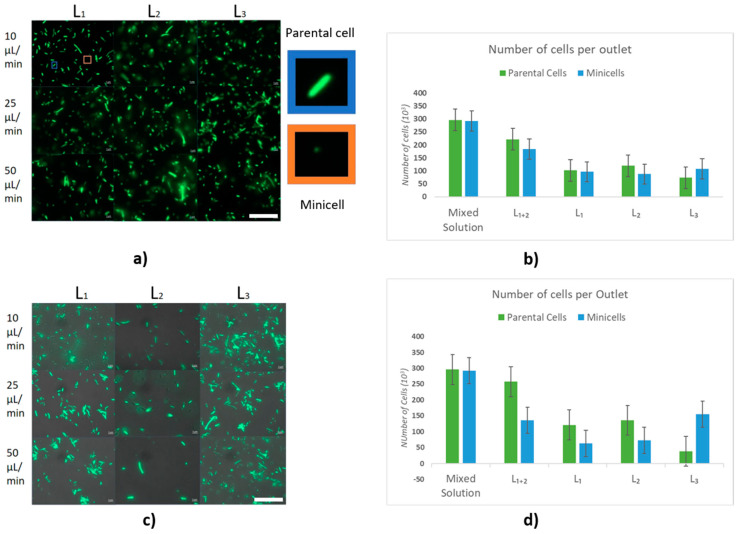
(**a**) Fluorescence microscopy image of cells at outlets L_1_, L_2_ and L_3_ of Chip A, flow rates 10, 25 and 50 µL/min respectively at rows a, b, and c; scale bar 25 µm. (**b**) Number of parental cells and minicells in the mixed and pure solutions for Chip A. (**c**) Cells at outlets L_1_, L_2_ and L_3_ of Chip B, flow rates 10, 25 and 50 µL/min respectively at rows a, b and c; scale bar 25 µm; (**d**) Number of parental cells and minicells in the mixed and pure solutions for Chip B.

Figure 5a,c show the fluorescence microscopy images of cells at three outlets and three flow rates in this design. Bar charts show the accumulation of minicells in the middle outlet (L_3_). The results in Figure 5b show that only 25% of the parental cells of the input suspension enter the middle outlet L_3_. However, L_3_ contains a higher amount of minicells compared to the side outlets L_1_ and L_2_. Based on Equation (5), the efficiency of Chip A, which is purely a comparative ratio between the solution accumulated in L3 and the solution prior to separation, is 32.3%, although the chip has no DLD effect. As said above, Chip A was designed for control experiments to assess the effect of cell morphology and has posts which are not tilted. However, due to their rod shape, the cells were dragged to L_1_ and L_2_, and the minicells went along the main flow to the L_3_. Outlet L_3_ included a different number of cells compared to the inlet; therefore, the separation efficiency is not zero but 32.3%. This implies that the cells moving through a simple symmetric lattice undergo a certain separation that allows the parental cells to move into the flanking outlets (L_1_ and L_2_), as observed in the control experiment already, while the minicells maintain their course towards the central outlet (L_3_). This effect could perhaps be due to the intermediate flow profile and diffusion considering the morphology of the cells. The standard deviation for sampling over different flow rates is measured by averaging over three samples of the same flow rate. The results showed no significant effect of flow rate on separation efficiency. The bar charts in Figure 5b,d are the values obtained for 50 µL/min flow rate. The other flow rates tested show virtually identical results (Figure S3). Similar tests were carried out on Chip B. The cells which accumulate in each outlet are shown in Figure 5d. Notably, the efficiency increases from 32.3% on Chip A to 75.5% on Chip B. As observed, the number of minicells in the middle outlet (L_3_) is more dominant compared to that in the side outlets L_1_ and L_2_. Moreover, the number of minicells that accumulate in L3 after the separation process is 3 times higher than that of parental cells. This supports our hypothesis of achieving a higher separation efficiency by reducing the critical diameter to 500 nm. It is observed that by optimizing the design parameters to have DLD separation, the efficiency increases by a factor of 2.3. This shows that the optimization of design, for instance changing parameters such as post size, gap and angle, significantly changes the separation efficiency.

## 4. Summary

In this work, we designed and fabricated two DLD PDMS microchips to separate cells which differ not only in size, but also in shape. We separated *E. coli* cells up to 10 µm long from spherical minicells with diameters between 450 and 500 nm. We optimized two microchips with a main channel including arrays of circular posts of 50 and 25 µm, spaced 15 and 2.5 µm, and with row shift angle of 0 and 1° (named Chips A and B). Indeed, we could separate parental, rod-shaped bacterial cells with a length of 1–10 µm from spherical minicells with a diameter of ~400–500 nm. For a mixed solution including both minicells and parental cells, we achieved high efficiency (75.5%) of separation with Chip B. Therefore, Chip B with DLD effects imposed by smaller posts and shifted rows provides higher separation efficiency and will be chosen for further scaled-up microchips integrated with the bioreactor. In order to study the efficiency of chips when separating by size only and the effects that the size of cells imposes on their flow profile, we tested the flow of pure wild-type cells as a control experiment. We observed that both designs show promising separation results. However, Chip A with larger posts provides more cells in the outlet. This is due to Chip A having zero Dc. We also observed that, in all the experiments, the rod-shaped cells have a flow trajectory towards the channel walls and side outlets due to their size being larger than the critical diameter of the designed chips, whereas the minicells are mostly collected in the middle outlet following the flow direction. We also studied the effect of shear on cell separation and pumped the cell solution in the chips at three different flow rates, i.e., 10, 25 and 50 µL/min. The results showed that changing the flow rate does not have a significant effect on the ratio of the minicells collected at the outlets and, consecutively, does not result in changes in the separation efficiency. Our results allow for a comprehensive understanding that will be used for future improvement in the field for multistage cell separation designs down to nanometer particles and for different bacteria. The multistage cell separation designs would allow for intermediate flow factors to be considered. They will also allow for more than one critical diameter for the separation, which could break down the separation stages into segments for a high-throughput solution after the separation process. For high-throughput separation and to collect cells of desired properties at a faster pace, higher flow rates are preferred. Our design facilitates the integration of such single-stage microchips with multiple chips having high-throughput capable systems and integration with bioreactors for further cell separation applications.

## Figures and Tables

**Table 1:** Geometrical design parameters for DLD microchips A and B.

Design Parameters	Chip A	Chip B
Post diameter, $D_{P}$ (µm)	50	25
Posts spacing, G (µm)	15	2.5
Posts row shift angle, *θ*	0°	1°

## Supplementary Materials

The following supporting information can be downloaded at: https://www.mdpi.com/article/10.3390/mi13030365/s1, Figure S1: Wild-type cells DLD flow through the post areas, Figure S2: Effect of the flow rates on separation efficiency, Figure S3: Microscopy images of inlet-to-outlet flow, Video S1: Cell flow—middle post area, Video S2: Cell flow—channel side posts.
