# Supplementary material for: Deterministic Lateral Displacement Microfluidic Chip for Minicell Purification
Source: Micromachines (Basel). 2022 Feb 25;13(3):365. doi: 10.3390/mi13030365 (PMC8951003; doi:10.3390/mi13030365)
Supplement: Supplementary file 1 [file micromachines-13-00365-s001.zip › micromachines-1601064-supplementary.pdf]

micromachines

Supplementary

Deterministic Lateral Displacement Microfluidic Chip for Minicell Purification

Ahmad Sherbaz [1,2], Büşra M. K. Konak [2], Pegah Pezeshkpour [1*], Barbara Di Ventura[2] and Bastian E. Rapp [1,3,4]

1 Laboratory of Process Technology, NeptunLab, Department of Microsystems Engineering (IMTEK), University of Freiburg, 79110 Freiburg im Breisgau, Germany 1; ahmad.sherbaz@neptunlab.org; Pegah.Pezeshkpour@neptunlab.org ; Bastian.Rapp@neptunlab.org
2 Signalling Research Centres BIOSS and CIBSS, Institute of Biology II, Faculty of Biology, University of Freiburg; barbara.diventura@biologie.uni-freiburg.de; busra.merve.kirpat.konak@biologie.uni-freiburg.de;
3 Freiburg Materials Research Center (FMF), University of Freiburg, 79104 Freiburg im Breisgau, Germany
4 FIT Freiburg Center of Interactive Materials and Bioinspired Technologies, University of Freiburg, 79110 Freiburg im Breisgau, Germany
* Correspondence: Pegah.pezeshkpour@neptunlab.org

Error! Reference source not found. represents the flow with red line and marks on the microscopy image for particles larger than critical diameter while the green line represents the particles smaller than critical diameter. On the right is the actual figure taken from channel of a Chip B with 50 µm post size and it can observe that the elongated bacterial cells are shifting on an upward DLD trajectory as predicted.

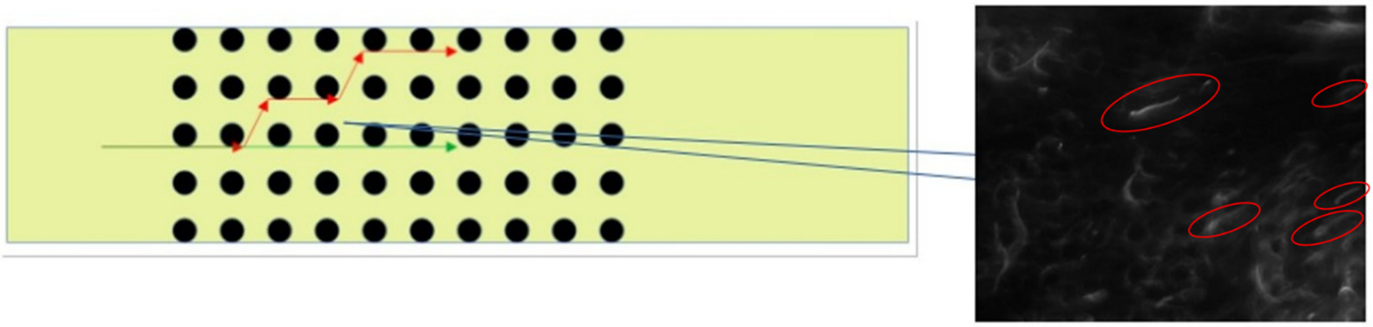

Figure S1. Wild-type cells DLD flow through the post areas.

In order to show that the flow rate does not affect the efficiency of chips, we tested different flow rates of 10 µl/min, 25 µl/min and 10 µl/min on both Chip A and B to see the effect of higher shear on the separation efficiency. The results proved that the efficiency is independent of the flow rate. The efficiency and standard deviation is shown in **Error! Reference source not found.**. However, higher flowrates lead to faster accumulation of cells which is of great benefit to clinical trials with high-throughput sample solutions.

Figure S2 shows the left to right flow of the solution at the top half of the horizontal main channel which is characterised by the parental cells moving towards the side channel, while the same is true for the bottom half. However, in the middle they following a bump mode.

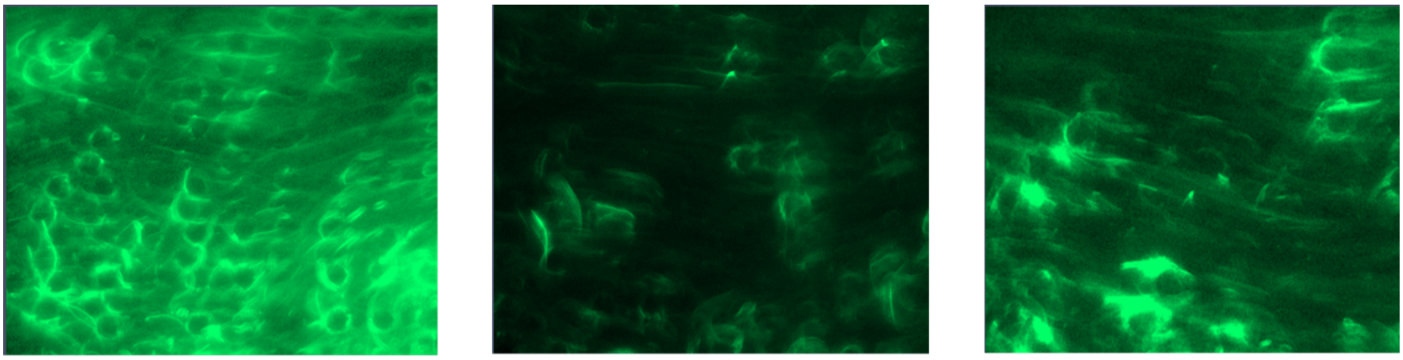

a)Top of the channel flow b)Middle of the channel flow c)Bottom of the channel flow

Figure S2. Microscopy images of the inlet-to-outlet flow (left to right) of the solution through the post area at a) top part of the channel, b) middle of the channel, and c) bottom part of the channel.

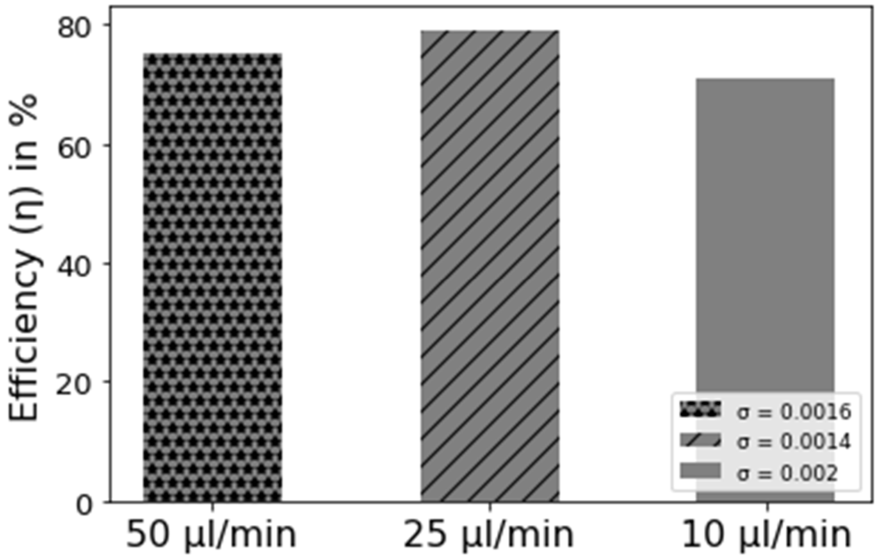

Figure S3. Effect of the flowrates on separation efficiency.
